# Cerium Oxide Nanoparticles/Polyacrylonitrile Nanofibers as Impervious Barrier against Viral Infections

**DOI:** 10.3390/pharmaceutics15051494

**Published:** 2023-05-13

**Authors:** Merna H. Emam, Reham S. Elezaby, Shady A. Swidan, Samah A. Loutfy, Rania M. Hathout

**Affiliations:** 1 Nanotechnology Research Center (NTRC), The British University in Egypt, Suez Desert Road, El-Shorouk City, P.O. Box 43, Cairo 11837, Egypt; 2 Department of Pharmaceutics and Industrial Pharmacy, Faculty of Pharmacy, Ain Shams University, Cairo 11566, Egypt; 3 Department of Pharmaceutics and Pharmaceutical Technology, Faculty of Pharmacy, The British University in Egypt, El-Shorouk City, P.O. Box 43, Cairo 11837, Egypt; 4 Virology and Immunology Unit, Cancer Biology Department, National Cancer Institute, Cairo University, Cairo 11796, Egypt

**Keywords:** polyacrylonitrile, nanofibers, cerium oxide, nanoparticles, adenovirus, face mask

## Abstract

Background: Using face masks is one of the protective measures to reduce the transmission rate of coronavirus. Its massive spread necessitates developing safe and effective antiviral masks (filters) applying nanotechnology. Methods: Novel electrospun composites were fabricated by incorporating cerium oxide nanoparticles (CeO_2_ NPs) into polyacrylonitrile (PAN) electrospun nanofibers that can be used in the future in face masks. The effects of the polymer concentration, applied voltage, and feeding rate during the electrospinning were studied. The electrospun nanofibers were characterized using SEM, XRD, FTIR, and tensile strength testing. The cytotoxic effect of the nanofibers was evaluated in the *Vero* cell line using the MTT colorimetric assay, and the antiviral activity of the proposed nanofibers was evaluated against the human adenovirus type 5 (*ADV-5*) respiratory virus. Results: The optimum formulation was fabricated with a PAN concentration of 8%, *w*/*v* loaded with 0.25%, *w*/*v* CeO_2_ NPs with a feeding rate of 26 KV and an applied voltage of 0.5 mL/h. They showed a particle size of 15.8 ± 1.91 nm and a zeta potential of −14 ± 0.141 mV. SEM imaging demonstrated the nanoscale features of the nanofibers even after incorporating CeO_2_ NPs. The cellular viability study showed the safety of the PAN nanofibers. Incorporating CeO_2_ NPs into these fibers further increased their cellular viability. Moreover, the assembled filter could prevent viral entry into the host cells as well as prevent their replication inside the cells via adsorption and virucidal antiviral mechanisms. Conclusions: The developed cerium oxide nanoparticles/polyacrylonitrile nanofibers can be considered a promising antiviral filter that can be used to halt virus spread.

## 1. Introduction

Viral infections have been a leading cause of morbidity and mortality globally and are one of the major reasons that lead to economic losses [1,2]. Several complications are caused by viral infections, including respiratory, thromboembolic, and cardiovascular diseases, which might be associated with secondary bacterial infections along with the viral infections [3]. Wearing face masks is recommended in the routine infection prevention and control (IPC) practices reported by the US Centers for Disease Control and Prevention (CDC) [4]. There is a huge need to develop advanced protective tools to control the speed of viral spread among people, reduce the need for hospital admission, and protect people’s lives, especially high-risk individuals.

Human adenoviruses (*ADVs*) can cause infectious outbreaks with serious clinical manifestations that might lead to death [5]. They are double-stranded DNA non-enveloped viruses that belong to the family Adenoviridae [6]. They can affect multiple human organs such as the gastrointestinal tract, respiratory tract, and ocular surface. Children and adults with immunodeficiencies are more susceptible to developing adenoviral infection [7,8].

Meanwhile, protective tools play a substantial role in the transmission of respiratory viral infections. Commercial face masks can partially prevent microbial particles from entering host cells. However, they are unable to inactivate the virus on reaching the mask [9]. For these reasons, there is a demand to develop antiviral filters that can be incorporated into commercial masks to provide specific protection against respiratory viruses.

Nanotechnology is considered a front-line method of fighting viruses [1] and a versatile tool that can provide great advances in the development of viral treatments and protective tools [10]. Among nanocarriers, nanofibers (NFs) exhibit distinctive characteristics, such as a high surface-area-to-volume ratio, remarkable mechanical strength, and, most significantly, being highly porous, possessing small pores [11]. In addition, they can capture particles smaller than 50 nm, which cannot be achieved using surgical face masks [12]. They are able to efficiently capture the smallest droplets carrying microbes, thus preventing them from causing infections [1]. They can be fabricated using several techniques, such as self-assembly, phase separation, template synthesis, centrifugal spinning, and drawing techniques [13]. The most preferred method for fabricating nanofibrous mats is electrospinning, owing to its low cost, simplicity, and process controllability [14].

The use of polymers with antiviral capabilities to eradicate viruses is a promising approach that can be used for fabricating protective tools [15,16]. Several methods have been suggested, such as using polymers with charged moieties, such as polyanions or polycations, and polymers with organic backbones, or adding tiny antiviral molecules, such as metal ions [17]. Polyacrylonitrile (PAN) is a thermoplastic synthetic polymer that possesses good mechanical properties as well as a high chemical [18] and thermal stability [19]. It has been incorporated in various fields including filtration media, biomedical textiles, and protective clothing [20]. PAN NFs have a great potential for use in face masks, owing to them possessing polar functional groups that have a high affinity towards particulate matter and therefore achieving a high removal efficiency and adequate air permeability [21]. PAN NFs provide a mask with favorable physicochemical and mechanical characteristics. Moreover, they had antiviral activity when previously tested against ADV-5, which is related to the polyanionic nature of the polymer [22]. Previously, they have been used for mask applications after fabrication and loading with biomaterials such as the angiotensin-converting enzyme-2 (ACE-2) receptor [22], a metal–organic framework (MOF) [23], metal and metal oxide nanoparticles (NPs), such as silver (Ag) [24,25], zinc oxide (ZnO), titanium dioxide (TiO_2_) [25], and copper oxide (CuO) NPs [26], graphene oxide [27], antibacterial agents such as N-halamine [28], and antiviral agents such as viroblock [19] and tetrahydropyrimidine (PTHP) [29]. Blank PAN NFs [30] and nylon 6-PAN composite nanofibrous mats [31] have been prepared before and tested for filtration applications. In addition, PAN—polyvinylidene fluoride (PVDF) composite NFs were fabricated and loaded with silver nitrate NPs [32].

The usefulness of nano-sized metal and metal oxide materials has been studied in the literature [33]. They are utilized for various antiviral, anticancer, antibacterial, catalytic, solar cell, sensor, as well as environmental protection applications [34,35,36,37,38,39,40,41]. Cerium oxide nanoparticles (CeO_2_ NPs) have attracted ever-increasing attention due to their extraordinary properties. They are promising pharmacological agents with remarkable biological activities against various viruses, bacteria, as well as tumors [38,42,43]. They were shown to exert antiviral activity against herpes simplex virus 1 (HSV-1), HSV-2, and influenza A virus subtype H1N1 [44]. Furthermore, Ag-modified CeO_2_ NPs were effective against OC43, human coronavirus, and RV14, human rhinovirus [38]. They were discovered to have the greatest potential for scavenging reactive oxygen species (ROS) among all metal oxide nanoparticles [42], which protects the cells from any oxidative stress exerted by pathogens [45].

Despite the fact that CeO_2_ NPs possess unique antimicrobial properties [38,42], they are not biocompatible with normal cells [46]. The incorporation of CeO_2_ NPs into PAN nanofibrous mats greatly enhances their biocompatibility as well as their antiviral properties, making this composite nanofibrous mat a promising one for antiviral protection.

The aim of this work was to develop a novel PAN nanofibrous composite membrane loaded with cerium oxide nanoparticles. This composite can be used in protective antiviral masks, being of small pore size and loaded with cerium oxide nanoparticles possessing proven antiviral activity. CeO_2_ NP-loaded PAN NFs were developed and characterized using a transmission electron microscope (TEM), a scanning electron microscope (SEM), Fourier-transform infrared spectra (FT-IR), an X-ray diffractometer (XRD), and a uniaxial tensile testing machine. The blank and composite NFs were evaluated for antiviral activity using molecular studies.

## 2. Materials and Methods

### 2.1. Materials

Polyacrylonitrile (PAN; average molecular weight = 150,000 g/mol) and dimethyl sulfoxide (DMSO; ACS reagent for analysis) were purchased from Sigma-Aldrich, St. Louis, MO, USA. N, N-Dimethyl formamide (DMF; ≥99%; laboratory reagent grade) was purchased from Fisher Chemical, Hampton, NH, USA. N, N-Dimethylacetamide (DMAc; analytical research grade) was purchased from Alpha Chemika, Andheri, India. Cerium oxide nanoparticles (CeO_2_ NPs) were purchased from NanoGate Company, Nasr City, Egypt. MTT (3-[4,5-dimethylthiazol-2-yl]-2,5-diphenyltetrazolium bromide) dye was purchased from Serva Electrophoresis GmbH, Heidelberg, Germany. 

### 2.2. Fabrication of PAN NFs and CeO_2_ NP-Loaded PAN NFs

PAN was dissolved in N, N-dimethylformamide (DMF) at concentrations of 6%, 8%, and 10%, *w*/*v* in closed vials to avoid bubble formation. The solutions were stirred overnight at 50 °C until complete dissolution [28]. Afterward, the prepared solutions were electrospun into NFs using an electrospinner (NANON-01A, MECC, Fukuoka, Japan). The electrospinning solutions were added to a plastic 5 mL syringe with an 18 G stainless-steel needle. Electrospinning was performed in varying operating regimes using feed rates of 0.6 to 1.4 mL/h, applied voltages of 26 to 29 KV, and a tip-to-target distance of 15 cm. The fabricated NFs were collected on aluminum foil covering a static plate collector covered in aluminum foil. All NFs were electrospun at room temperature with a humidity of 50–55% and dried at 70 °C for 24 h to evaporate any residual solvent. Depending on the SEM micrographs of the fabricated NFs, the optimum PAN NFs were selected for loading with CeO_2_ NPs. For fabricating CeO_2_ NP-loaded PAN NFs, CeO_2_ NPs (0.25%, *w*/*v*) were first dispersed in DMF. Thereafter, the dispersion was added slowly to a PAN solution (8%, *w*/*v*) in DMF and stirred overnight at 50 °C to obtain a homogenous mixture. Finally, the resultant mixture was electrospun into composite NFs at a feed rate of 0.5 mL/h, an applied voltage of 26 kV, and a 15 cm distance between the needle and plate collector. The electrospinning conditions of some of the prepared NFs are listed in Table 1.

### 2.3. Characterization of CeO_2_ NPs

#### 2.3.1. Transmission Electron Microscope (TEM)

The size and morphology of the CeO_2_ NPs were investigated using transmission electron microscopy (TEM) (JEOL JEM-1010, Tokyo, Japan). A drop of the CeO_2_ NP suspension was placed over a carbon-coated copper grid and air-dried for 5 min at room temperature [47]. The sample was examined at an accelerating voltage of 200 kV. TEM images were processed using ImageJ software to calculate the average size of the NPs. 

#### 2.3.2. Polydispersity Index (PDI) and Zeta Potential Determination

The PDI and zeta potential of the CeO_2_ NPs were measured using a Zetasizer (Nano ZS, Malvern Instruments Ltd., Malvern, UK) with dynamic light scattering (DLS) and Doppler velocimetry techniques, respectively [48]. A freshly prepared CeO_2_ NP dispersion was diluted appropriately with deionized water at 25 °C via probe sonication to produce a homogenous dispersion with a measurable scattering intensity. The sample was injected into a universal folded capillary cell with gold electrodes at both ends.

### 2.4. Characterization of PAN, and CeO_2_ NPs-Loaded PAN NFs

#### 2.4.1. Scanning Electron Microscope (SEM)

The surface morphologies of the electrospun nanofibrous mats were examined using a scanning electron microscope (SEM) (Quattro S, Thermo-Scientific, Waltham, MA, USA) with an acceleration voltage of 5–30 kV at room temperature. The samples were carefully cut into appropriate sizes and placed on a specific grid. SEM images were processed to calculate the average NF diameters using ImageJ software by examining 25 randomly chosen NFs from each fabricated sample. 

#### 2.4.2. Fourier-Transform Infrared Spectra (FT-IR)

The chemical compositions of the CeO_2_ NPs, PAN NFs, and CeO_2_-PAN NFs were assessed using Fourier-transform infrared spectra (Bruker Vertex 70, Bremen, Germany). Before the analysis of the samples, the background used was the spectrum of air. Spectroscopic analysis was performed with IR fingerprints recorded between 4000 and 400 cm^−1^ at a spectral resolution of 4 cm^−1^ using transmittance modes via the attenuated total reflection (ATR) crystal at room temperature. For each measurement, 32 scans were performed.

#### 2.4.3. X-ray Diffractometer (XRD)

The crystalline structures of the CeO_2_ NPs, PAN NFs, and CeO_2_-PAN NFs were detected using an X-ray diffractometer (XRD) (Shimadzu 7000, Kyoto, Japan) with CuKα radiation (λ = 1.5418 Å). The scans were performed over an angular range of 0°–80° (2θ) with a 0.02° step size and a 0.5 s time per step. XPertHighscore Plus software was used for data analysis.

#### 2.4.4. Mechanical Strength

The mechanical strengths of the prepared blank PAN and CeO_2_-PAN NFs were determined using a uniaxial tensile testing machine (Autograph AG-X series (table-top type), Shimadzu, Japan) equipped with a load cell of 20 N. Mechanical parameters, breaking strain (%), maximum displacement (mm), and tensile stress (KPa) were measured for the tested NFs.

### 2.5. Cell Culture and Cytotoxicity Assay

The *Vero* cell line was obtained from the ATCC (American tissue culture collection, Manassas, VA, USA) and used as a model for normal cells. *Vero* cells were grown in Dulbecco’s Modified Eagle Medium (DMEM, Lonza, Bornem, Belgium) supplemented with 10% fetal bovine serum (FBS, Gibco, Waltham, MA, USA), 2% penicillin/streptomycin (Lonza, Belgium), 1% sodium pyruvate (100 mM, Lonza, Belgium), and 1% L-glutamine (200 mM, Lonza, Belgium). The cells were incubated at 37 °C, 5% CO_2_, and 85–95% humidity. The cellular toxicities of the CeO_2_ NPs, PAN (8%, *w*/*v*), and CeO_2_-PAN nanofibrous mats were investigated in *Vero* cells using MTT (3-[4,5-dimethylthiazol-2-yl]-2,5-diphenyltetrazolium bromide) dye after 48 h of incubation with the investigated preparations [49,50]. The cytotoxic effect of the CeO_2_ NPs was evaluated using 2-fold serial dilutions starting from 30 to 1.875 µg/mL, while, the cellular toxicity of the CeO_2_-PAN NFs was determined after the treatment of the cells with concentrations of 15 and 30 µg of CeO_2_ NPs. The assay was performed in triplicate. The cytotoxicity concentration (CC_50_) value of the CeO_2_ NPs was determined using the GraphPad^®^ prism software v.8.0.2. The following equation was used to calculate the viability of cells (%) in relation to the control wells [22,51]:

$$\mathrm{Cell}\;\mathrm{viability}\;(\%)=\frac{A_{\mathrm{test}}}{A_{\mathrm{control}}}\times 100$$

where (A_test_) is the mean absorbance of the tested sample, and (A_control_) is the mean absorbance of the control sample.

### 2.6. Antiviral Assay

The antiviral activity of non-toxic concentrations of PAN and CeO_2_-PAN NFs was studied against *human adenovirus type 5 (ADV-5)* (*ATCC VR-5*), which was provided by the ATCC (American tissue culture collection, USA) and propagated into normal *Vero* epithelial cells using a quantitative real-time polymerase chain reaction (qPCR) assay.

#### 2.6.1. Infectivity Assay

*ADV-5* was grown in *Vero* cells and examined daily using an inverted microscope (Axio Observer 5, Carl Zeiss, Jena, Germany) until achieving 80–90% of cytopathic effect (CPE). Afterward, the viral load was determined using qPCR. The standard curve was built using 10-fold serial dilutions of standard *ADV-5* (*ATCC VR-5*) (10–10^6^ copies/mL) [6].

#### 2.6.2. Titration of ADV-5 DNA in Cell Culture

Viral titration was performed by seeding 2 × 10^4^ *Vero* cells per well into a 96-well tissue culture plate. The plate was incubated for 24 h under standard conditions of 37 °C and 5% CO_2_. Afterward, the cells were infected with a 2-fold serial dilution of an *ADV-5* stock of a known viral load and incubated for 48 h until 80–90% cell lysis was observed. The quantitation of viral load was performed using a qPCR assay [51], and the IC50 was determined according to the highest viral dilution that killed 50% of the infected cells.

#### 2.6.3. Quantitation of ADV-5 DNA in Cell Culture

This was performed using a qPCR assay after nucleic acid extraction and a qPCR assay following the same procedures in our recently published reports [22,52].

#### 2.6.4. Antiviral Activity of CeO_2_ NP-Loaded PAN NFs

This was assessed against *ADV-5* on the safest materials (blank PAN NFs and 15 µg CeO_2_ NPs), and then, they were subjected to two antiviral mechanisms, the viral adsorption and viral replication/virucidal mechanisms.

##### Adsorption Mechanism

A 6-well plate was seeded with 5 × 10^5^ cells per mL and incubated under standard conditions. The cells were treated with our materials after 24 h. After 24 h, they were infected with IC50 of *ADV-5* stock and incubated for another 24 h under standard conditions. The virus culture was subjected to three cycles of freezing and thawing for the quantification of the viral load using a qPCR assay [53].

##### Virucidal Mechanism

A 6-well plate was seeded with 5 × 10^5^ cells per mL and incubated at 37 °C and 5% CO_2_. After 24 h, the cells were infected with a mixture of IC_50_ of the virus and tested materials that were previously incubated for 1 h at 4 °C. The plate was furtherly incubated for 24 h, and cells were subjected to a qPCR assay, as previously described for the adsorption mechanism [53].

Two controls were run in each of the two assays. The first one was a control cell, which was an untreated well. The second one was a viral control, which was an untreated infected well. All plates were then subjected to a qPCR assay to measure viral load. Two other controls were run in each qPCR assay, a positive control (*ADV-5* of known copies/mL) and a negative control (water) [51].

### 2.7. Statistical Analysis

All tests were conducted in triplicate, and the results were expressed as means ± standard deviation (SD). Statistical analysis of the means of the two groups was performed using Student’s *t*-test with *p* < 0.05 using GraphPad^®^ prism v.8.0.2. Furthermore, statistical analysis of the means of three groups or more was performed using one-way analysis of variance (ANOVA) with *p* < 0.05 using the same software, in which the ANOVA was followed by Tukey’s multiple comparison test to compare all the pairs.

## 3. Results and Discussion

### 3.1. Transmission Electron Microscope (TEM)

**Figure 1:**
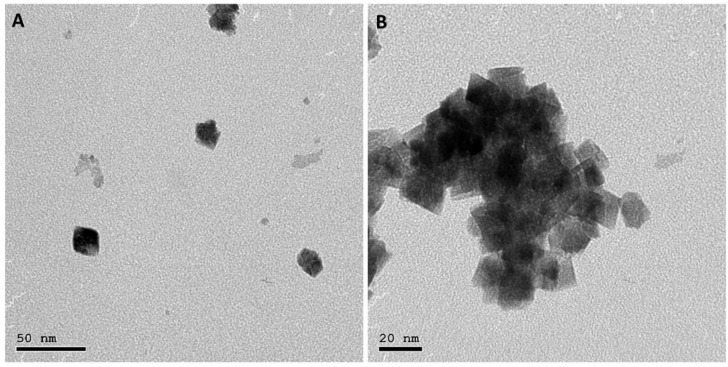
TEM images of CeO_2_ NPs with different scales: (**A**) 50 nm and (**B**) 20 nm.

TEM imaging was used to examine the size and shape of the CeO_2_ NPs. As shown in Figure 1, the NPs were uniform in size with a cubical shape. The size of the NPs was determined to be 15.8 ± 1.91 nm.

### 3.2. Polydispersity Index (PDI) and Zeta Potential of CeO_2_ NPs

The zeta potential of the CeO_2_ NPs was recorded as −14.1 ± 0.001 mV, which indicates that the NPs are considered to be unstable in a colloidal dispersion and show a high probability of the particles’ agglomeration. The PDI of the CeO_2_ NPs was recorded as 0.9985 ± 0.002, which confirms the high liability for aggregations. Hence, the incorporation of CeO_2_ NPs into the PAN nanofibrous mat was warranted in order to decrease the aggregation of NPs.

### 3.3. Scanning Electron Microscope (SEM)

**Figure 2:**
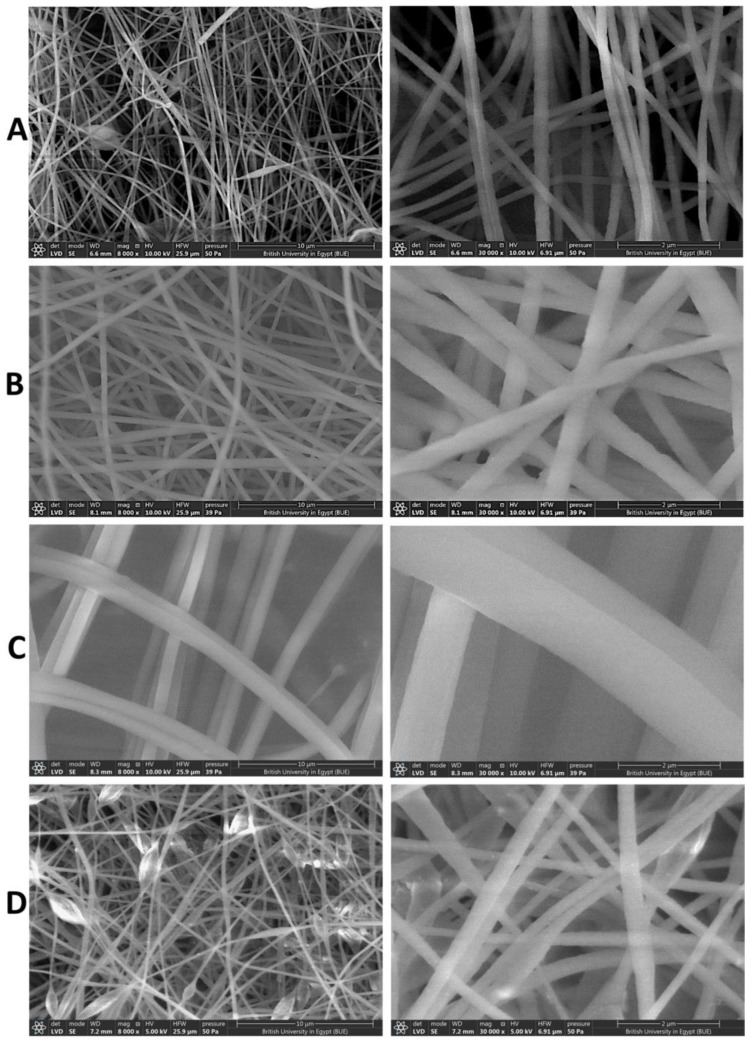
SEM images of (**A**) blank PAN NFs (F1, 6%), (**B**) blank PAN NFs (F2, 8%), (**C**) blank PAN NFs (F3, 10%), and (**D**) CeO_2_ NPs-PAN NFs (F4), with magnification power of 8000× (left panel) and 30,000× (right panel).

**Figure 3:**
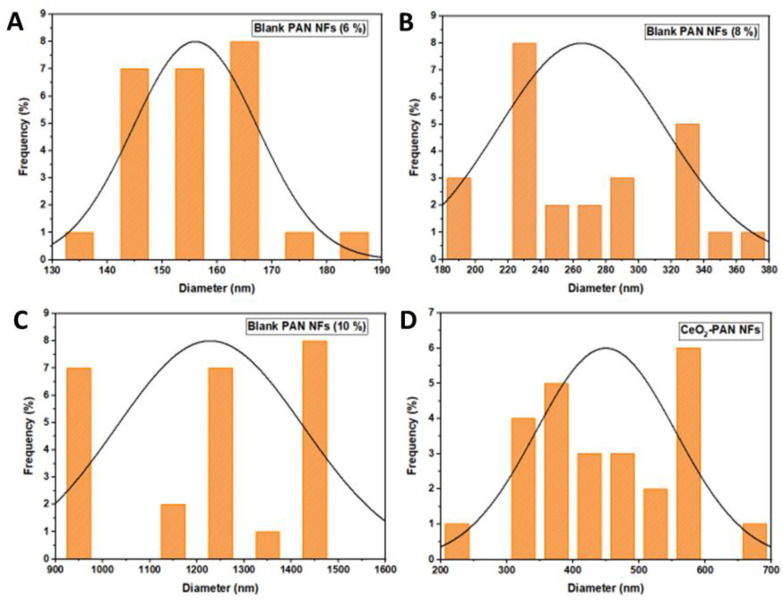
Diameter distributions of (**A**) blank PAN NFs (6%), (**B**) blank PAN NFs (8%), (**C**) blank PAN NFs (10%), and (**D**) CeO_2_ NPs-PAN NFs.

An SEM was intensively used to examine the surface morphology of the NFs with different concentrations of PAN (6, 8, and 10%), as shown in Figure 2. The PAN NFs (8%) (Figure 2A) displayed an accepted morphological structure of relatively uniform, continuous, and interconnected NFs with no formed beads. They were selected as the optimum blank NFs owing to the presence of beads in PAN 6% NFs (Figure 2B) and the large diameter, poor, and deformed NFs of PAN 10% (Figure 2C). This is in agreement with Huang et al., who observed that optimum PAN NFs were fabricated from PAN 8% compared with PAN 5% and 10% [28]. As demonstrated in Figure 3, the average diameters of the NFs were 156.04 ± 11 nm, 385.11 ± 50 nm, and 1227.88 ± 198 nm for PAN 6%, PAN 8%, and PAN 10% NFs, respectively. The diameter of the produced NFs is directly proportional to the concentration of PAN. Similar findings were obtained in a study conducted by Huang et el., in which the fibers’ diameters increased with an increasing concentration of PAN [28]. Increasing the polymer concentration limits the stretching of the solution while electrospinning because of the increase in the viscosity [54]. Additionally, the solvent vaporizes faster in the higher concentration of the polymeric solution, resulting in producing NFs with large diameters [54]. The average diameters of the nanofibers decreased significantly (*p* < 0.05) with an increasing applied voltage during electrospinning (Table S1, and Figures S1 and S2). Pillay et al. reported that increasing the voltage results in decreasing the nanofibers’ diameters [55]. Some SEM micrographs demonstrated that nanofibers formed with elongated beads or knot-like structures (Figure S1). This is in line with the findings of Korycka et al., who reported that the voltage affects both the development of beaded fibers and the beads’ size [56]. The average diameter of the nanofibers increased significantly (*p* < 0.05) with an increasing feeding rate (Table S1, and Figures S1 and S2). This agrees with the findings of Jabur et al., who fabricated polyvinyl alcohol, polyvinylpyrrolidone, and nylon 6 nanofibers and reported that increasing the feeding rate was associated with the increasing diameters of the nanofibers [57]. It can be distinctly observed that after the CeO_2_ NPs’ encapsulation, uniform and beads-free NFs were obtained, as seen in Figure 2D. This implies that incorporating the NPs did not result in changes in the NFs’ structural uniformity or bead formation. Interestingly, several swollen parts were seen in the micrographs, which indicate the encapsulation of the CeO_2_ NPs into the NFs. It was observed that incorporating NPs into nanofibrous mats increased the diameter of the NFs significantly (*p* < 0.05) [26,58]. After loading CeO_2_ NPs into the selected concentration of PAN (8%), the NFs’ average diameters were 449.64 ± 105 nm.

### 3.4. Fourier-Transform Infrared Spectra (FT-IR)

**Figure 4:**
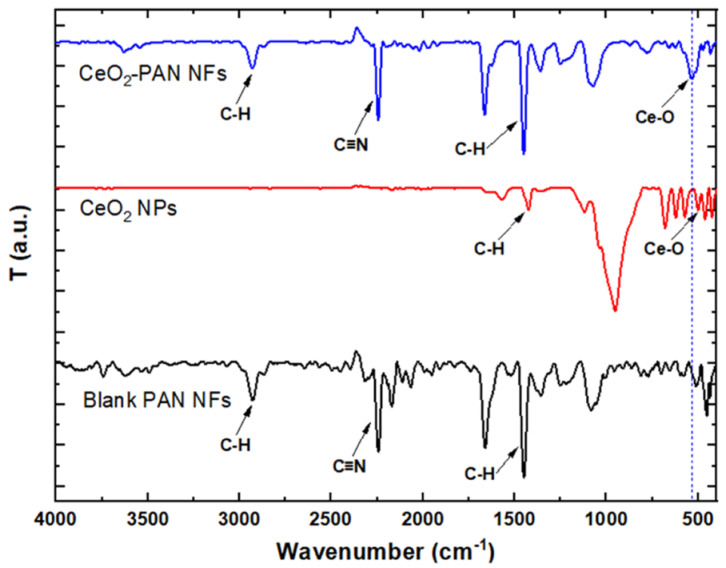
FT-IR spectra of blank PAN NFs (F2, PAN 8%), CeO_2_ NPs, and CeO_2_-PAN NFs (F4).

As shown in Figure 4, the spectrum of the blank PAN NFs showed characteristic peaks at 2933 cm^−1^, 2241 cm^−1^, and 1454 cm^−1^, which are determined as C-H stretching, -C≡N stretching, and -C-H bending peaks, respectively [58,59,60]. The FT-IR spectrum of the CeO_2_ NPs showed a peak at 500 cm^−1^, corresponding to the Ce-O vibration in the CeO_2_ NPs [61,62]. The FT-IR spectrum of the CeO_2_-PAN NFs revealed characteristic peaks at 2933 cm^−1^, 2241 cm^−1^, and 1454 cm^−1^, which are determined as C-H stretching, -C≡N stretching, and -C-H bending peaks, respectively. Interestingly, the peak at 500 cm^−1^ in the spectrum which corresponds to the Ce-O peak of the CeO_2_ NPs was shifted to 533 cm^−1^ after incorporating the CeO_2_ NPs, as demonstrated in the FT-IR spectrum of the CeO_2_-PAN NFs. Additionally, no peaks were newly formed nor disappeared, which indicates the absence of chemical interactions between PAN NFs and CeO_2_ NPs [62] and that the polymeric solution of PAN and CeO_2_ NPs was just physically mixed together.

### 3.5. X-ray Diffraction (XRD)

**Figure 5:**
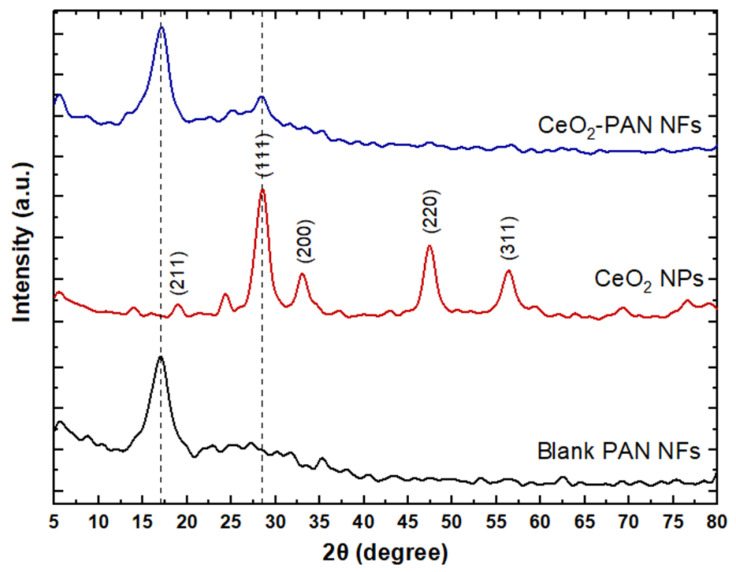
XRD patterns of blank PAN NFs (F2, PAN 8%), CeO_2_ NPs, and CeO_2_-PAN NFs (F4).

The fabricated nanofibrous mats were analyzed using XRD to investigate their crystalline properties. The diffraction patterns are shown in Figure 5. The diffraction peaks of the CeO_2_ NPs at 2θ = 28.5°, 33°, 47.4°, and 56.3° correspond to the (111), (200), (220), and (311) crystal planes, respectively, having a cubic CeO_2_ structure with the Inorganic Crystal Structure Database (ICSD) reference code 01-071-4199, along with a low-intensity peak at 2θ = 19° indexed as (211) for cubic Ce_2_O_3_ based on ICSD 01-072-6357 [61,63]. The XRD pattern of PAN showed a semicrystalline structure with a sharp diffraction peak superimposed on a hump at 2θ = 17° [62,64]. A comparison of the XRD of the blank PAN NFs with that of the CeO_2_-PAN NFs showed the appearance of a strong peak at 2θ = 28° (111) as well as the disappearance of all the other CeO_2_ NPs peaks, which could be attributed to the destruction of the NPs’ crystal lattice. This could be explained by the encapsulation of the NPs in the nanofibrous mats.

### 3.6. Standard Uniaxial Tensile Test

The breaking strain (%), maximum displacement (mm), and tensile strain (%) of the electrospun NFs were measured to assess the mechanical properties of the NFs before and after incorporating CeO_2_ NPs. Interestingly, the incorporation of CeO_2_ NPs into the PAN NFs showed no significant change in the maximum displacement values of the NFs (*p* > 0.05) compared with blank PAN NFs. As displayed in Table 2, the breaking strain and tensile strain values of the NFs decreased significantly (*p* < 0.05) after incorporating the CeO_2_ NPs. These findings may indicate that the NPs agglomerated and represented stress-concentrating regions in the nanofibrous mats, which could have a negative impact on their mechanical properties. Previous studies demonstrated a decrease in NFs’ strengths after incorporating metal and metal oxide NPs [65,66].

### 3.7. Cytotoxicity Assay

**Figure 6:**
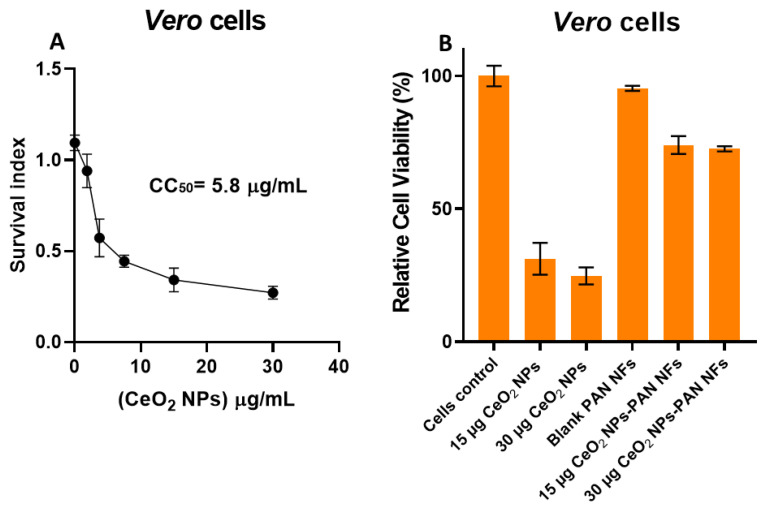
(**A**) CC50 of CeO_2_ NPs in Vero cells; (**B**) cell viability (%) via MTT assay of CeO_2_ NPs and CeO_2_-PAN NFs in Vero cells. Results are represented as means ± SD.

The cytotoxic effects of various concentrations of CeO_2_ NPs (30, 15, 7.5, 3.75, and 1.875 µg/mL) revealed that its CC50 (the highest dilution of the tested material that kills 50% of the cells) in *Vero* cells was detected at a concentration of 5.8 µg/mL (Figure 6). The illustrated results reveal that the blank PAN NFs were safe in Vero cells and demonstrated 95% cellular viability associated with no significant difference from the control (*p* > 0.05). Meanwhile, for the different concentrations of CeO_2_ NPs, there was a significant decrease (*p* < 0.05) in the survival index with the increasing concentration; however, there was no significant change between 15 µg and 30 µg CeO_2_ NPs. Moreover, the results show that the viability of CeO_2_ NP-treated cells increased significantly (*p* < 0.05) from 25–31% to 74–95% after the incorporation of the NPs into the PAN NFs. There was no recorded significant difference in the cellular viability of the 15 µg CeO_2_-PAN NFs and 30 µg CeO_2_-PAN NFs. The biocompatibility of the CeO_2_ NPs may be attributed to the antioxidant and anti-inflammatory activity of the NPs, which have been reported in previous studies [39,44,67]. They have the ability to scavenge ROS and reactive nitrogen species (RNS) present inside cells, which is essential for cellular viability and biological activity [44,67]. They are considered an anti-inflammatory agent, as they suppress inflammatory pathways, thus successfully reducing the oxidative stress and cellular structural damage caused by the tested materials [44]. The antioxidant and antiapoptotic properties of the developed CeO_2_ NPs [64] are attributed to their reversible transfer from a reduced state into an oxidized state [43,67].

### 3.8. Antiviral Assay

**Figure 7:**
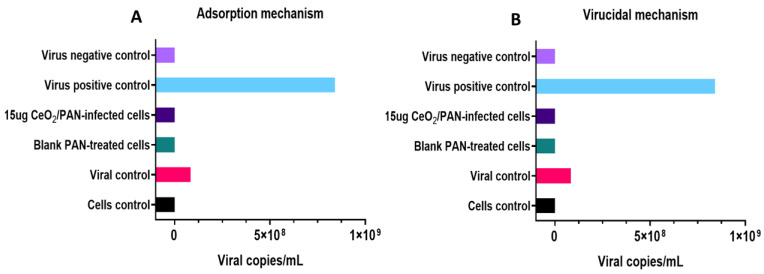
Antiviral assay of blank PAN (F2, 8%) and 15 µg CeO_2_-PAN NFs against ADV-5 via both (**A**) adsorption and (**B**) virucidal mechanisms.

The viral titration of *ADV-5* (the dilution of the virus that can kill 50% of cells) was found to be 10^5^ copies/mL, which is equivalent to a 1:100 dilution of the viral stock that was used in the antiviral assay. The results of the performed experiments using qPCR are presented in Tables S2 and S3. As illustrated in Figure 7, the results show that blank PAN (8%, *w*/*v*) and 15 µg CeO_2_-PAN NFs exert their antiviral activity via adsorption and virucidal mechanisms, as evidenced in the undetected viral copies using a qPCR assay. The tested samples were able to inhibit the entry of virus particles into host cells, as well as prevent viral infectivity via virucidal mechanisms after incubation with the virus for 1 h.

Regarding the antiviral activity of CeO_2_-PAN NFs via the adsorption mechanism, it was revealed to be attributed to the negative charges that are present on the surface of PAN [68], which was able to interact with viral host receptors, thus preventing its attachment to host cells and its ability to enter and infect host cells [17,69]. This approach has been previously reported in HIV infection [22]. With regard to the virucidal antiviral mechanism, it may be accredited to the interaction of our tested composite NFs with viral proteins, specifically the hexon protein, which was amplified using a qPCR assay [22,52]. Further insight analysis based on molecular and protein levels is still needed to determine the impact of our materials on other viral proteins that play a major role in viral replication.

Previous studies have proposed CeO_2_ NPs as an antiviral [39,44,67] as well as pro-oxidant agent against viral infections [43,44]. The reported pro-oxidant activity of CeO_2_ NPs is due to their ability to generate ROS and produce cell damage in pathological conditions [43,44].

Moreover, some approaches have been suggested in previous studies to clarify the mechanisms of the antiviral activity of CeO_2_ NPs. Previous work reported that CeO_2_ NPs exhibit activity against OC43 seasonal human coronavirus, and human rhinovirus 14. This was attributed to the physical interaction of CeO_2_ NPs with the OC43 envelope, which disrupts its lipid bilayer integrity [38]. Moreover, the chemical interaction of CeO_2_ NPs with the protein shell of RV14 denatures the receptor-binding proteins, inactivates the virion [38], and prevents viral infection.

## 4. Conclusions

Face masks provide a physical barrier that can prevent microbial emissions from diseased people. Antiviral polyacrylonitrile nanofibers (PAN NFs) that were successfully electrospun and loaded with cerium oxide nanoparticles (CeO_2_ NPs) were developed. The nanofibrous layers are accountable for capturing and eliminating microbial particulates. This role was augmented by the antiviral properties of CeO_2_ NPs as well as PAN NFs. The face mask filter action was demonstrated in the significantly higher cellular viability after incorporating the NPs into the nanofibrous membrane. Moreover, it demonstrated significant antiviral activity against the *ADV-5* virus, which reveals its superiority over commercial face masks. The proposed filter can be incorporated into commercial face masks or sewn into washable fabric masks to cut down expenses, offering potent and cost-effective personal protection against respiratory viruses during pandemics and outbreaks. Accordingly, the PAN/CeO_2_ nanofibrous composite is considered a potential antiviral face mask filter that can significantly limit the transmission of respiratory viruses.

## Figures and Tables

**Table 1:** Examples of fabricated PAN and CeO_2_-loaded PAN NFs.

Formula Code	PAN Concentration (%, *w*/*v*)	CeO_2_ NP Concentration (%, *w*/*v*)	Voltage (kV)	Feed Rate (mL/h)	Distance between Needle and Collector (cm)
F1	6	_	28	0.6	15
F2	8	_	26	1.4	15
F3	10	_	29	1	15
F4	8	0.25	26	0.5	15

**Table 2:** Mechanical properties of electrospun PAN (F2, 8%) and CeO_2_-PAN NFs (F4).

	PAN NFs	CeO_2_-PAN NFs
Breaking strain (%)	25.6 ± 1.41	7.33 ± 1.6
Max. displacement (mm)	12.7 ± 3.29	12.1 ± 2.69
Tensile strain (%)	395.5 ± 1.5	325.63 ± 2.12

## Data Availability

Data Would be available upon request.

## Supplementary Materials

The following supporting information can be downloaded at: https://www.mdpi.com/article/10.3390/pharmaceutics15051494/s1, Figure S1: SEM images of electrospun PAN NFs of (A) F1, (B) F2, (C) F3, (D) F4, (E) F5, (F) F6, (G) F7, (H) F8, (I) F9, (J) F10, and (K) F11; Figure S2: Diameter distribution of NFs of the electrospun NFs F1, F2, F3, F4, F6, F7, F8, F9, F10, and F11; Table S1: Composition of different formulae for studying solution and spinning conditions of PAN NFs, NFs morphology and NFs’ diameters; Table S2: qPCR assay of ADV-5-treated Vero cells with the tested materials via virucidal mechanism; Table S3: qPCR assay of ADV-5-treated Vero cells with the tested materials via adsorption mechanism.
