# Supplementary material for: Cerium Oxide Nanoparticles/Polyacrylonitrile Nanofibers as Impervious Barrier against Viral Infections
Source: Pharmaceutics. 2023 May 13;15(5):1494. doi: 10.3390/pharmaceutics15051494 (PMC10224416; doi:10.3390/pharmaceutics15051494)
Supplement: Supplementary file 1 [file pharmaceutics-15-01494-s001.zip › pharmaceutics-2354904-supplementary.pdf]

SUPPLEMENTARY MATERIALS

Cerium Oxide nanoparticles/Poly acrylonitrile Nanofibers as Impervious Barrier Against Viral Infections

TableS1. Composition of different formulae for studying solution and spinning conditions of PAN NFs, NFs morphology and NFs' diameters.

The studied factor	Formula code	PAN conc (%, w/v)	Solvent	Voltage (KV)	Feed rate (mL/h)	Distance (cm)	Morphology	Nanofibers' diameter (nm)
**PAN conc.**	F1	6	DMF	26	1.4	15	Nanofibers formed with elongated beads	159.81 ± 36
	F2	8	DMF	26	1.4	15	Continuous, beads-free Nanofibers	385.11 ± 50
	F3	10	DMF	26	1.4	15	Nanofibers formed with less beads	694.69 ± 69
**Solvent**	F4	8	DMF	28	1	15	Nanofibers formed with elongated beads	262.85 ± 47
	F5	8	DMSO	28	1	15	Semi, discontinued fibers formed vertically with low spinnability	No nanofibrous mat formed to measure the nanofibers' diameter
	F6	8	DMAc	28	1	15	Nanofibers formed with beads and some bundles	446.48 ± 101
**Distance**	F7	8	DMF	28	1	11	Nanofibers	414.31 ± 90

**between needle & collector**							formed with some bundles and knot-like structures	
	F8	8	DMF	28	1	13	Nanofibers formed with elongated beads	333.14 ± 47
	F4	8	DMF	28	1	15	Nanofibers formed with elongated beads	262.85 ± 47
**Voltage**	F9	8	DMF	26	1	15	Nanofibers formed with elongated beads	323.37 ± 50
	F4	8	DMF	28	1	15	Nanofibers formed with elongated beads	262.85 ± 47
	F10	8	DMF	30	1	15	Nanofibers formed with elongated beads	189.16 ± 44
**Feeding rate**	F11	8	DMF	26	0.6	15	Nanofibers with knot-like structures	385.11 ± 37
	F9	8	DMF	26	1	15	Nanofibers formed with elongated beads	323.37 ± 50
	F2	8	DMF	26	1.4	15	Continuous, beads-free Nanofibers	385.11 ± 50

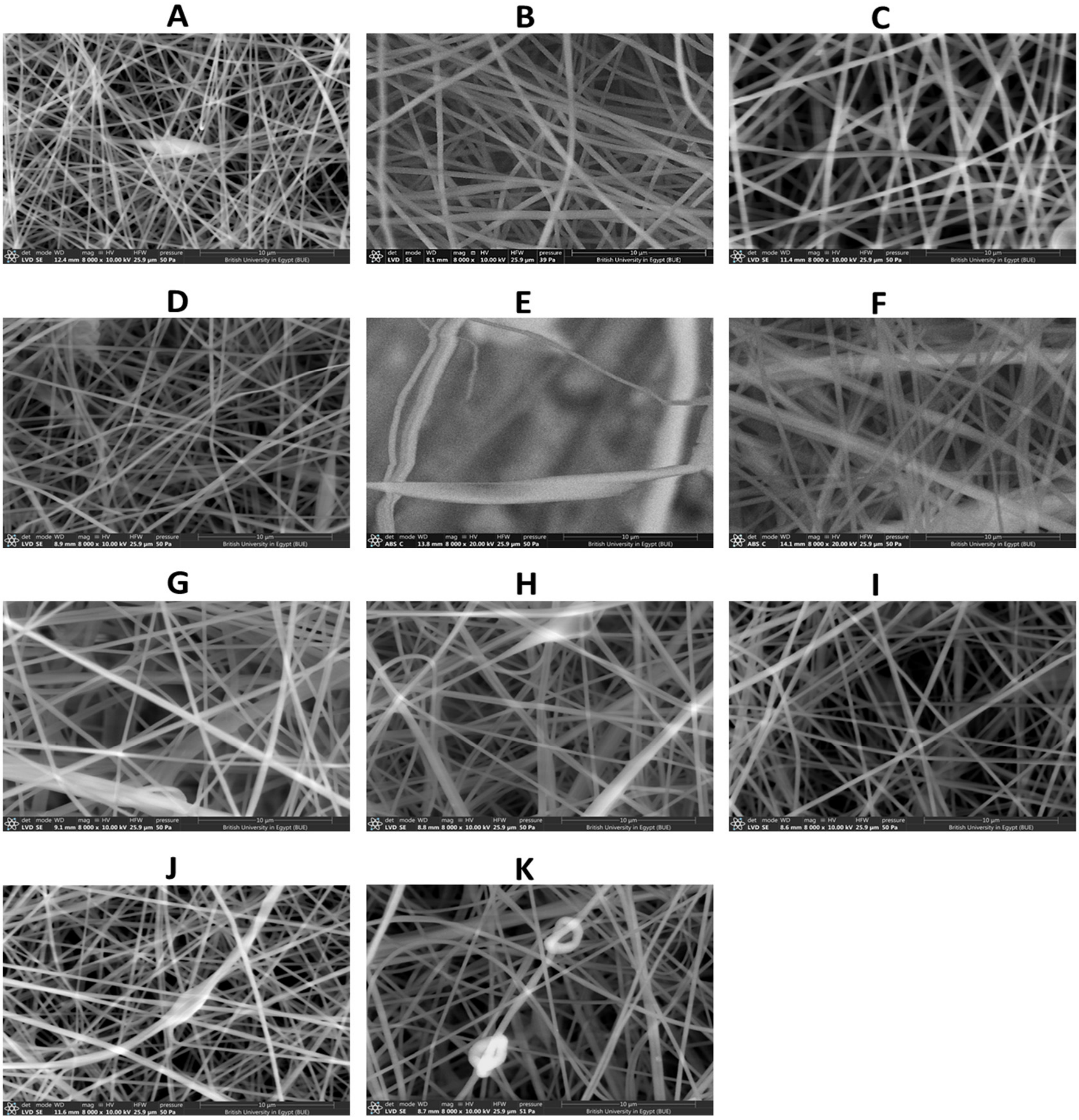

Figure S1. SEM images of electrospun PAN NFs of (A) F1, (B) F2, (C) F3, (D) F4, (E) F5, (F) F6, (G) F7, (H) F8, (I) F9, (J) F10, and (K) F11.

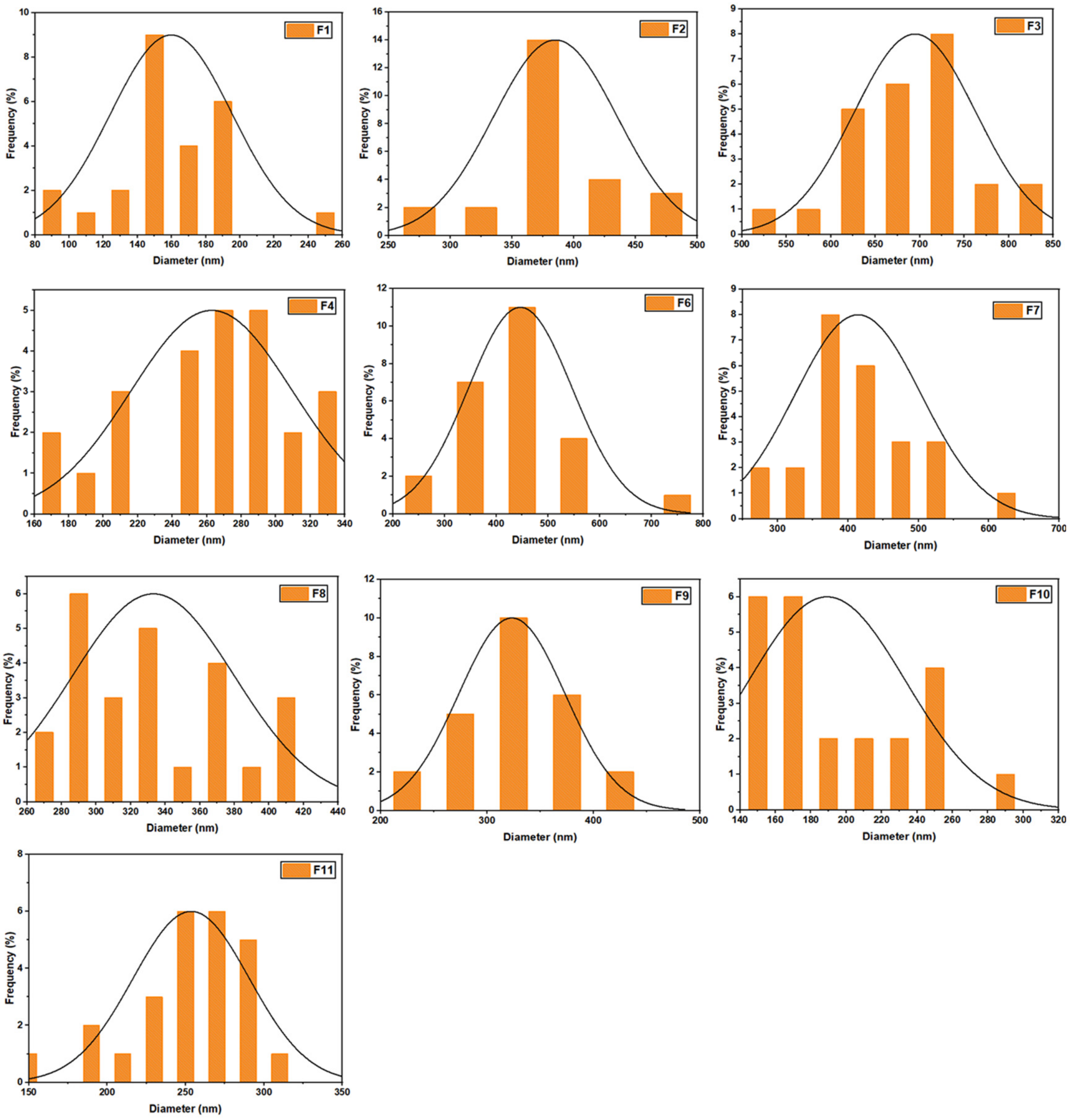

Figure S2. Diameter distribution of NFs of the electrospun NFs F1, F2, F3, F4, F6, F7, F8, F9, F10, and F11.

Table S2. qPCR assay of *ADV-5*-treated *Vero* cells with the tested materials via virucidal mechanism

ADV-5	**CT[a]**	**Copies/mL**
Blank PAN NFs	Under detection	Under detection
PAN NFs-15 µg	Under detection	Under detection
Untreated Vero cells (cell control)	Under detection	Under detection
Positive cells infected with AdV5 (viral control)	12.9	$8.3x10^{7}$
Positive control	17	$2.5x10^{6}$
Negative control	Under detection	Under detection

[a] CT: threshold cycle.

Table S3. qPCR assay of *ADV-5*-treated *Vero* cells with the tested materials via adsorption mechanism

ADV-5	**CT[a]**	**Copies/mL**
Blank PAN NFs	Under detection	Under detection
PAN NFs-15 µg	Under detection	Under detection
Untreated Vero cells (cell control)	Under detection	Under detection
Positive cells infected with AdV5 (viral control)	27.6	$1.62x10^{4}$
Positive control	22	$2.7x10^{4}$
Negative control	Under detection	Under detection

[a] CT: threshold cycle.
